# MC-SleepNet: Large-scale Sleep Stage Scoring in Mice by Deep Neural Networks

**DOI:** 10.1038/s41598-019-51269-8

**Published:** 2019-10-31

**Authors:** Masato Yamabe, Kazumasa Horie, Hiroaki Shiokawa, Hiromasa Funato, Masashi Yanagisawa, Hiroyuki Kitagawa

**Affiliations:** 1 Graduate School of Systems and Information Engineering, University of Tsukuba, Tsukuba, Japan; 2 Center for Computational Sciences, University of Tsukuba, Tsukuba, Japan; 3 International Institute for Integrative Sleep Medicine, University of Tsukuba, Tsukuba, Japan

**Keywords:** Biomedical engineering, Computational models

## Abstract

Automated sleep stage scoring for mice is in high demand for sleep research, since manual scoring requires considerable human expertise and efforts. The existing automated scoring methods do not provide the scoring accuracy required for practical use. In addition, the performance of such methods has generally been evaluated using rather small-scale datasets, and their robustness against individual differences and noise has not been adequately verified. This research proposes a novel automated scoring method named “MC-SleepNet”, which combines two types of deep neural networks. Then, we evaluate its performance using a large-scale dataset that contains 4,200 biological signal records of mice. The experimental results show that MC-SleepNet can automatically score sleep stages with an accuracy of 96.6% and kappa statistic of 0.94. In addition, we confirm that the scoring accuracy does not significantly decrease even if the target biological signals are noisy. These results suggest that MC-SleepNet is very robust against individual differences and noise. To the best of our knowledge, evaluations using such a large-scale dataset (containing 4,200 records) and high scoring accuracy (96.6%) have not been reported in previous related studies.

## Introduction

Sleep in mice consists of three stages: WAKE, non-REM (non-rapid eye movement sleep), and REM (rapid eye movement sleep). These stages play different roles in the lives of mice, and can be identified by inspecting electroencephalogram (EEG) and electromyogram (EMG) signals. Identifying the sleep stages of mice from their EEG and EMG signals (hereinafter referred to as “sleep stage scoring”) is one of the most fundamental inspections in sleep research. For example, the symptoms of sleep disorders and the effects of sleeping pills can easily be verified by analyzing the rates/transitions of sleep stages.

Traditionally, sleep stage scoring has been conducted through manual inspection by human experts; therefore this task requires considerable time and specialized knowledge about sleep in mice. Thus, manual sleep stage scoring is a serious bottleneck in sleep research. To resolve this problem, several automated sleep stage scoring methods have been proposed^1–8^. Unfortunately, these methods have the following limitations and shortcomings, when used in practical sleep research.

First, these methods cannot achieve the accuracy level required for practical research use. Generally, the inter-rater agreement rate of the manual sleep stage scoring results in mice is reported to be approximately 95% and greater^9^. To replace manual scoring, automated methods need to achieve the same accuracy level. The existing methods cannot achieve this level^1–8^ (the state-of-the-art method achieves nearly 95%^4,6^).

Second, their robustness against individual differences and noises has not been adequately verified. In practical sleep research environments, sleep records of various mice need to be analyzed (a record is a pair of EEG and EMG time-series signals recorded for approximately 24–72 hours from a mouse during one measurement). In addition, these records often include noise, such as motion artifacts and power supply noises. Therefore, the scoring methods should have robustness against such noises. However, the existing methods are generally evaluated using rather small-scale datasets, such as those consisting of several dozen mice records, which often contain only clear signals. Therefore, their robustness has not been adequately verified, and it is unclear whether these methods are actually effective in practical sleep research.

To address this problem, this study aims to propose a novel automated sleep stage scoring method that can achieve more accurate and more robust results than the existing methods and to verify its practical performance by experiments using a large-scale dataset.

Our proposed method is named “MC-SleepNet”, and it employs two types of deep neural networks: a convolutional neural network (CNN) and long short-term memory (LSTM)^10,11^.

CNN is a neural network model used for locating effective features of EEG and EMG signals and extracting them. Several studies have recently employed CNNs for biological signal processing, such as waveform detection for ECG/EEG signals of humans^12–15^, and showed that CNNs can extract effective features in biological signals. By using a CNN, MC-SleepNet can automatically extract more effective features than the hand-engineered features often used in conventional automated scoring methods. For example, it is very difficult to manually hand-craft filters to capture features to identify individual differences and noise. In contrast, CNNs can locate these features through the training phase, which will lead to improvements in the overall scoring accuracy and robustness.

LSTM is a type of recurrent neural network and is an effective model for long time-series data. In contrast to typical recurrent neural networks, the LSTM model has a “forget gate” structure for adjusting the period to maintain the information. Since the forget gate widens the usage of past information, LSTM can handle long time-series data well. This property will be helpful for modeling the sleep cycles and sleep stage transition rules.

In addition, an expansion of the LSTM model, bidirectional LSTM (bi-LSTM), has also been proposed^16^. This model consists of two LSTM layers with different roles. One layer processes the data in the order of time scale similar to the typical LSTM, and the other layer processes it in a reverse order (from the future to the past). By employing two different LSTM layers, bi-LSTM can consider the future states and the past states. MC-SleepNet uses bi-LSTM.

Another feature of this study is the performance evaluation of MC-SleepNet using a large-scale dataset containing the sleep records of 4,200 phenotypically wild-type healthy mice through a large-scale genetic screen^17^, which are used in practical sleep research. Besides, some of these records include considerable noise. Using this large-scale, real-world dataset, we verified the robustness of MC-SleepNet against individual differences and noise as well as its accuracy. To prove the advantages of MC-SleepNet, we compare its performance with that of the existing automated sleep-scoring methods.

## Sleep Stages of Mice

The sleep/wake state of mice can be divided into three sleep stages: WAKE, non-REM, and REM. Each stage is characterized by its own features in EEG and EMG signals as follows. In particular, the peak frequency of EEG signals and the amplitude of EMG signals are the key features.

### Wake

**Figure 1:**
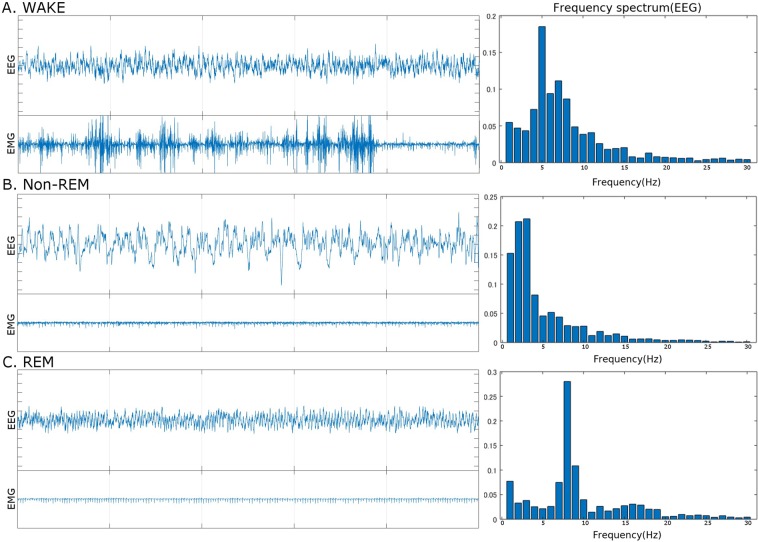
Examples of EEG/EMG signals in each stage. (**A**) WAKE, (**B**) non-REM, and (**C**) REM.

In WAKE stages, mice are awake or drowsy, and both their brains and bodies are active. Therefore, the EEG signals show mixed frequencies, and the amplitude of EMG signals tends to be large (Fig. 1A).

### Non-Rem

In non-REM stages, brains exhibit cortical synchronization, and bodies are resting. Thus, the peak EEG frequencies become lower and the amplitude of EEG signals becomes higher than in the WAKE stages, and the amplitude of EMG signals tends to be smaller (Fig. 1B). Non-REM is the major sleep stage and accounts for approximately more than 90% of sleep.

### Rem

In REM stages, brains are as active as in WAKE stages, whereas their bodies exhibit “REM atonia” such that the EMG amplitudes reach their lowest levels (Fig. 1C). Note that healthy mice in REM stages remain completely stationary, except for breathing, eye movement, and several muscle twitches.

Generally, the sleep stages change cyclically, and several rules govern sleep stage transitions. For example, it is known that REM stages occur at regular intervals during sleep, and persist for the duration of several tens of seconds to several minutes. Furthermore, REM stages do not occur immediately after WAKE stages in healthy mice. Understanding these transition rules will be helpful for improving the scoring accuracy.

Manual sleep stage scoring typically consists of two steps: biological signal acquisition and sleep stage identification. In the biological signal acquisition step, experts measure time-series EEG and EMG signals from the brain surface and neck muscles of mice for hours. The measured signals are divided into constant time intervals of 4 to 20 seconds, which are often called epochs. In the sleep stage identification step, the experts visually check the frequency components and amplitudes of the EEG and EMG signals, and assign one sleep stage label to each epoch. They essentially decide the label based on both the dominant features in each epoch and the sleep stage transition rules.

## Results

This section introduces our proposed sleep stage scoring method, “MC-SleepNet”, and presents the experimental evaluation results using a large-scale dataset.

### MC-SleepNet

MC-SleepNet is an automated sleep stage scoring method that identifies the sleep stage of each epoch. Here, we describe the structure and processing of MC-SleepNet through an implementation example that we used in the experiment. The hyperparameters were decided by referencing to deep learning model for scoring human sleep stages^18^. MC-SleepNet consists of three phases: signal preprocessing, feature extraction, and scoring.

#### Signal preprocessing

As mentioned above, in EMG signals, the amplitude is more informative than its frequency-domain features for identifying sleep stages. For this reason, we preprocess EMG signals using a moving root mean squared filter. We adopt a filter width of 1 second (Eq. 1) to emphasize its amplitude.

1 $${y}_{t}=\frac{1}{{F}_{s}-1}\sqrt{\mathop{\sum }\limits_{i}^{{F}_{s}}\,{({x}_{t-i})}^{2}},$$

where *x*_*t*_ and *y*_*t*_ are the input and output signals at time *t*, respectively, and *F*_*s*_ is the sampling frequency of the input signal.

MC-SleepNet does not preprocess EEG signals; rather, it analyzes the measured signals directly. EEG signals have both frequency-domain features and time-domain features. Therefore, we do not employ preprocessing, such as fast Fourier transform (FFT) to avoid disturbing CNN feature extraction.

In the preprocessing phase, EEG and preprocessed EMG signals are split into 20-seconds epochs, and a series of epochs is input to the feature extraction phase.

#### Feature extraction

**Figure 2:**
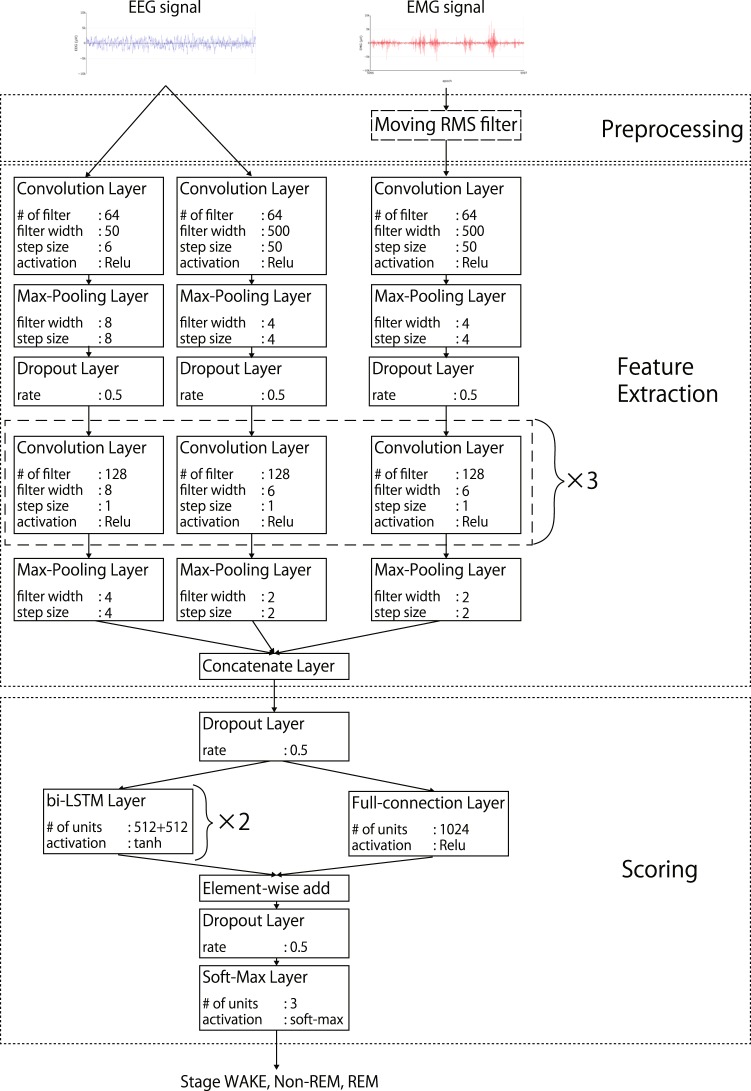
Structure of MC-SleepNet. MC-SleepNet uses eight types of layers: convolution, max-pooling, dropout, concatenate, element-wise add, bi-LSTM, full-connection, and softmax layers. The parameters of each layer are illustrated in the boxes.

MC-SleepNet uses a CNN to locate effective features automatically from EEG and EMG signals (Fig. 2). The feature extraction module utilizes three CNN blocks to extract different types of features in EEG and EMG signals.

In CNNs, the width of a filter is closely related to the main frequency of features extracted by the filter. For example, wide filters are effective in extracting low-frequency features such as the amplitude of signals, whereas narrow filters are good for locating high-frequency features. Thus, to extract both features from EEG signals, MC-SleepNet combines two CNN blocks that have wide and narrow filters, respectively. In contrast, EMG signals lack high-frequency feature components due to the preprocessing phase; therefore, MC-SleepNet uses only one CNN block of wide filters for EMG signals.

#### Scoring

The scoring module consists of a bi-LSTM block, fully connected (FC) block, and softmax layer. This module models the relationship between the extracted features and sleep stages considering sleep stage transition rules.

These blocks have different roles in scoring. The bi-LSTM block maintains the information of 25 consecutive epochs including the target epoch, and models the sleep stage transitions. In contrast, the FC block focuses on the target epoch, and detects “isolated” epochs whose stages are different from the consecutive neighboring epochs. MC-SleepNet uses two layered bi-LSTM blocks. Each block has 1024 LSTM units and uses half of these units to model the forward sleep stage transitions and the other half to model the backward transitions. The FC blocks has one FC layer that has 1024 artificial neurons.

The outputs of both the bi-LSTM and FC blocks are input into the softmax layer. This layer consists of three artificial neurons, and calculates the certainty of each of assigned stages (WAKE, non-REM, and REM). Finally, the scoring module outputs the most likely stage label.

#### Training

The training of MC-SleepNet consists of two steps: pretraining and fine-tuning. The main objective of the two-step training approach is to optimize a different module of MC-SleepNet in each step. The pretraining step will mainly optimize the feature extraction (CNN) module, while the fine-tuning step will optimize the scoring module of MC-SleepNet.

In the pretraining step, the scoring module (Fig. 2) is temporarily replaced with a softmax layer, which plays the same roles as the original scoring module. Then, we optimize the parameters of the feature extraction module by typical backpropagation. The loss function is categorical cross entropy, which is shown as follows.

2 $$L(Y,\hat{Y})=-\frac{1}{N}\mathop{\sum }\limits_{i}^{N}\,{{\bf{y}}}_{{\bf{i}}}\,\log \,{\hat{{\bf{y}}}}_{{\bf{i}}},$$

where *Y* and $\hat{{Y}}$ are sets of scored and actual sleep stage label vectors: **y** and $\hat{{\bf{y}}}$. They are three-dimensional vectors whose elements represent the certainty level of each sleep stage. *N* is the number of training samples. To reduce the effect of imbalanced training samples, we conduct undersampling to equalize the numbers of epochs for each stage. By conducting undersampling in the pretraining step, the feature extraction module can locate appropriate features of each sleep stage.

In the fine-tuning step, the softmax layer is replaced by the original scoring module. Then, the entire system is trained again using the same training dataset, and the parameters are optimized by backpropagation using the same loss function 2.

In our experiments, the pretraining and fine-tuning steps were repeated 10 and 20 times for each training dataset, respectively. These training processes were conducted using the Adam method^19^ with learning rates of 1*e* − 4 and 1*e* − 6 and batch sizes of 100 and 10. The reason why we used the smaller learning rate in the fine-tuning step is to prevent overfitting.

#### Rescoring

Although MC-SleepNet has sufficient performance for practical use, the recall for the REM stage is relatively lower than that for the other stages (Table 1). This could cause a problem in sleep research, especially in studies related to REM sleep in mice.

**Table 1:** Scoring performance on large-scale and small-scale datasets.

Scoring Methods	WAKE		Non-REM		REM		Acc.	*κ* stat.
	Rec.	Prec.	Rec.	Prec.	Rec.	Prec.		
**on large-scale dataset of 4,200 mice**								
**MC-SleepNet**	98.1%	**97.8%**	**95.8%**	97.3%	80.1%	**90.1%**	**96.7%**	**0.94**
MC-SleepNet + rescoring	**98.6%**	97.0%	94.4%	**97.8%**	**89.5%**	77.9%	96.3%	0.94
Random forest	95.9%	95.0%	94.0%	94.1%	76.9%	84.0%	94.1%	0.89
MC-SleepNet (noisy records)	94.8%	98.5%	97.5%	92.5%	75.7%	86.8%	95.0%	0.91
**on small-scale dataset of 14 mice**								
**MC-SleepNet**	**99.3%**	**98.6%**	93.9%	**99.6%**	**99.5%**	62.6%	**96.4%**	**0.94**
Random forest	95.9%	92.6%	94.3%	95.6%	73.9%	**88.9%**	94.0%	0.89
FASTER^2^	89.5%	—	94.2%	—	78.4%	—	91.1%	—
MASC^4^	95.5%	97.3%	**94.7%**	96.9%	94.0%	65.8%	95.0%	0.91
LSTM model^5^	96.2%	94.8%	95.1%	95.8%	82.1%	85.2%	94.9%	0.91

The relatively low recall for the REM stage could be due to different factors. The first factor is “isolated” REM epochs, where stage labels of their neighboring epochs are not REM. These epochs tend to be given the same stage labels as neighboring epochs. Another factor is the imbalanced sleep stage distribution. Generally, REM stages are rare in the all records. Consequently, in the training process, MC-SleepNet builds stricter criteria for REM stages than other sleep stages, and tends to hesitate to output REM stage labels. To address this problem, we developed a rescoring model as an optional method of MC-SleepNet, which carefully examines the possibility of REM stages.

In the rescoring method, we collect epochs that are given non-REM labels by MC-SleepNet and whose non-REM certainty values are lower than 95% and re-examine those epochs by the rescoring model (please refer to the “REM scoring by MC-SleepNet” section for details). This model has almost the same architecture as MC-SleepNet, except for the scoring module. It omits the bi-LSTM block from the original MC-SleepNet such that the model can output stage labels that are different from those of neighboring epochs more easily. The model was trained with epochs that met the above conditions. Therefore, the imbalance problem of training samples was alleviated.

### Evaluation with the large-scale dataset

We verified the scoring accuracy of MC-SleepNet using a large-scale dataset containing sleep records of 4,200 mice. This dataset is associated with the label dataset which contains “correct” sleep stage labels for all the epochs. These sleep stage labels were obtained through manual sleep stage scoring by human experts. Therefore, we can use the labeled dataset for training MC-SleepNet and validating the accuracy of the outputs of MC-SleepNet. In the accuracy validation, we measured the agreement rates of the outputs of MC-SleepNet with the “correct” labels.

Our sleep record dataset includes many noisy records. Therefore, the robustness of MC-SleepNet against individual differences and noise can also be evaluated with the dataset.

To compare the performance of MC-SleepNet with the state-of-the-art method, we measured the performance of the random forest model. It is known that the random forest model can handle large-scale datasets with reasonable computational costs. Please refer to the Methods section for more details of the dataset and random forest model.

In this experiment, we conducted five-fold cross validation. The 4,200 records were randomly split into five partitions: four partitions (3,360 records) were used as training samples, and the remaining partition (840 records) was used as test data. The experimental result (Table 1) shows that MC-SleepNet can score sleep stages with an accuracy of 96.6%, which is significantly higher than that of the random forest model (*p* < 0.01: paired t-test). In addition, its *κ* statistic is 0.91; therefore, the scoring results of MC-SleepNet and experts match very well^20^.

In addition, this result shows that the rescoring model improved the recall for the stage REM by approximately 9%. Although the overall accuracy and recall for the non-REM stage decreased slightly, the risk of missing REM epochs was greatly resolved. Therefore, the rescoring model will be effective, especially in research focusing on REM sleep.

**Figure 3:**
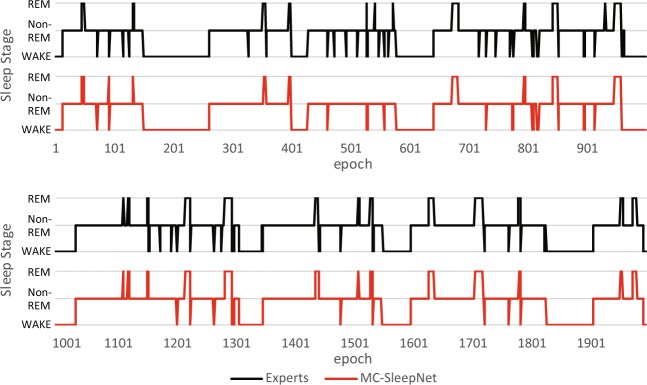
Example of sleep stage scoring result by MC-SleepNet.

An example of the scoring results is shown in Fig. 3. The horizontal axis shows a sequence of epochs, and the vertical axis corresponds to the three sleep stages. The orange and blue lines represent the scoring results of MC-SleepNet and human experts, respectively. This figure shows that both scoring results are very close to each other and almost consistent. These experimental results suggest that MC-SleepNet can achieve high accuracy in sleep stage scoring.

As we mentioned, the dataset contains many noisy sleep records from a number of mice. Thus, these results strongly suggest that MC-SleepNet is very robust against individual differences and noise. In the next section, we directly analyze the robustness against noise.

### Evaluation with noisy records

To directly analyze the robustness of MC-SleepNet against noise, we apply MC-SleepNet that was trained in the above experiment to noisy records and check the scoring accuracy. Note that noisy records were identified by human experts, and four records were chosen for this analysis.

As shown in Table 1, although the accuracy decreases from 96.6% to 95.0%, MC-SleepNet can score sleep stages with sufficient accuracy even if the sleep records include considerable noise. Comparing this result with the case of the whole dataset, MC-SleepNet tends to output non-REM stage labels more frequently. Therefore, the recall of the WAKE and REM stages and the precision of the non-REM stage decreased. The four records contain several types of noise that have low-frequency components such as motion artifact noise (0.5–2 Hz). This type of noise might be extracted as a delta-wave, causing an incorrect scoring.

### REM scoring by MC-SleepNet

**Figure 4:**
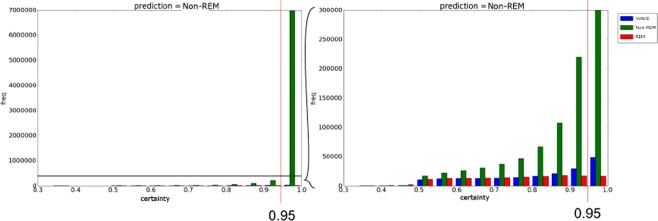
Histogram showing distribution of stages labeled as Non-REM.

Table 2 shows the confusion matrix of MC-SleepNet for the case of the large-scale dataset. Figure 4 plots the histogram showing the distribution of non-REM certainty values of epochs which are finally given non-REM stage labels by MC-SleepNet. (Their actual stage labels are shown in colors). The horizontal axis corresponds to the non-REM certainty values, and the vertical axis shows the frequencies. The right-hand graph magnifies the frequency range up to 300000 of the left-hand histogram. We can make the following observations.

- Table 2 suggests that most of incorrectly scored REM epochs (REM epochs but scored as other stages by MC-SleepNet) are given non-REM labels.
- Figure 4 suggests that most of the true non-REM epochs have very high non-REM certainty values (more than 95%).

**Table 2:** Confusion matrix for large-scale dataset.

		MC-SleepNet		
		WAKE	Non-REM	REM
Expert	WAKE	14087311	315295	8187
	Non-REM	233156	12800870	116645
	REM	39181	243181	1136074

Based on these observations, we collect epochs that are given non-REM labels by MC-SleepNet and whose non-REM certainty values are lower than 95% and re-examine those epochs by the aforementioned rescoring model.

### Comparison with other existing methods

We compare the scoring accuracy of MC-SleepNet with that of other existing methods: the FASTER, MASC, and LSTM models. The experimental setting is the same as that described above, except for the number of training iterations. The pretraining and fine-tuning steps were repeated 100 and 200 times in this experiment. In addition, oversampling was conducted to equalize the numbers of epochs for each stage. Unfortunately, these existing methods have a high computational cost for the training process. For this reason, we use another small-scale dataset used in the existing studies^4,5^. This dataset contains extremely clear sleep records obtained from 14 mice. Thus, the models do not have to locate/extract complicated noise features. Note that the numbers of training/testing samples (epochs) for MC-SleepNet are smaller than those for other existing methods. Since MC-SleepNet uses longer biological signals including past and future epoch, we could not use some epochs near the start and end of the measurement.

In this experiment, we conducted seven-fold cross validation and used 12 records as training samples. After the training process, MC-SleepNet achieved a scoring accuracy of 96.4% and kappa statistic of 0.94, which are higher than those of the existing methods (Table 1). This result proves that MC-SleepNet outperforms the existing methods.

## Discussion

**Figure 5:**
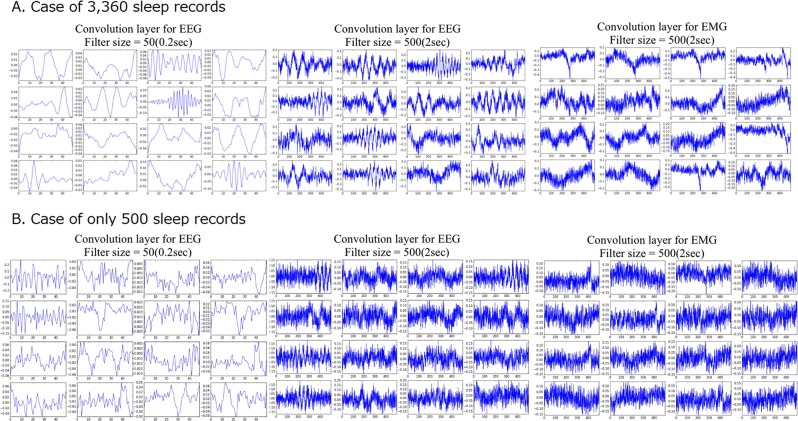
Example of extracted features by feature extraction module of MC-SleepNet. (left) EEG features extracted by narrow CNN. (center) EEG features extracted by wide CNN. (right) EMG features.

The greatest feature of MC-SleepNet is the use of a CNN in the feature extraction phase. As mentioned above, the CNN can locate effective features for sleep stage scoring. To verify the located features, we illustrate several features extracted by the feature extraction module of MC-SleepNet (Fig. 5A). These figures show that MC-SleepNet optimizes the filters to extract several frequency components in EEG signals and the instantaneous amplitude of EMG signals. These features are also used in manual sleep stage scoring. Therefore, we can state that MC-SleepNet can locate the effective features for sleep stage scoring through the training process. Interestingly, MC-SleepNet extracted several features that are not directly related to sleep stages. Specifically, some filters were optimized to extract waves that are neither theta waves nor delta waves. These waves might be related to individual differences and noise in EEG signals. By using such features, MC-SleepNet has achieved the high scoring accuracy and high robustness against individual differences and noise.

Due to these advantages, MC-SleepNet is effective, particularly in the large-scale sleep research. The high accuracy and robustness become more valuable as the number of sleep records to score increases. In addition, its scoring process is NOT time-consuming (approximately 15 s per 1-day sleep record). This is another advantage in large-scale research.

### Limitations

MC-SleepNet achieved more practical scoring than existing methods; however, it still has some practical limitations.

#### Cost for training

Although deep learning techniques enhance the scoring accuracy and robustness, they also cause some practical problems in the training process. First, to locate the effective features, MC-SleepNet requires a vast amount of training samples. Figure 5B illustrates the features in the case of only 500 sleep records used for training (note that the scoring accuracy was only 80.5% in this case). This result suggests that the feature extraction module of MC-SleepNet was not sufficiently optimized and they could not locate the effective features. To optimize MC-SleepNet to achieve a high scoring accuracy, a vast amount of training samples is required, making application of MC-SleepNet difficult in some situations.

In addition, the computational cost for the training process is another problem. For example, it took over 160 hours to optimize MC-SleepNet for the large-scale dataset in our experiment using a high-performance computer. This results suggest that the researchers who cannot afford to use a high-performance computer cannot optimize MC-SleepNet for their datasets. The difference in measurement environments can affect the scoring accuracy, which may be one of the factors that the users of MC-SleepNet need to be aware of.

#### Width of scoring epoch

Scoring mice sleep/wake with 20-second epochs tends to miss very short events such as transient arousals. This results in larger values of average “episode duration” statistics in general (which describes how long individual episodes of WAKE/non-REM/REM state last on average), as compared with scoring with 4-second or 10-second epochs. However, in our experience, relative changes in sleep/wake statistics and the recall in detecting such changes induced by, for example, genetic mutations such as Sleepy, Dreamless^17,21^, and orexin knockout^22^, are unaffected by scoring epoch lengths from 4–20 seconds. In addition, MC-SleepNet can be readily modified to accommodate shorter epoch lengths. Needless to say, an entirely new set of large-scale, high-quality training dataset with shorter epoch lengths will be needed.

## Methods

All of the procedures were conducted in accordance with the Guidelines for Animal Experiments of the University of Tsukuba and were approved by the Institutional Animal Care and Use Committee of University of Tsukuba (Approved protocol ID # 18-164).

### Dataset

Two datasets were used to evaluate the performance of MC-SleepNet. Both datasets contain the EEG and EMG signals split into 20-second epochs, and are associated with the label datasets containing “correct” sleep stage labels for all the epochs. The biological signals were obtained from wild-type healthy mice, and their “correct” sleep stages were scored by 13 experts from the International Institute for Integrative Sleep Medicine, University of Tsukuba. Their averaged inter-rater reliability and its standard deviation are 98.5% and 1.3%, respectively. Note that each biological signal record was inspected by one expert and his/her scoring results were used as “correct” sleep stages.

The datasets are different in the number of epochs and the clarity of the contained sleep records. The large-scale dataset contains 4,200 sleep records (35,700,000 epochs, where WAKE, non-REM and REM stages account for 49.7%, 45.4% and 4.9% of the total epochs, respectively), most of which include noise such as motion artifacts and power supply noises. Therefore, the robustness against the individual differences and noise of MC-SleepNet can easily be evaluated using this dataset. In contrast, the small dataset contains only 14 sleep records (238,000 epochs, where WAKE, non-REM and REM stages account for 42.6%, 52.7% and 4.7% of the total epochs, respectively), which are extremely clear. Thus, the records in this dataset can be scored relatively easily.

**Figure 6:**
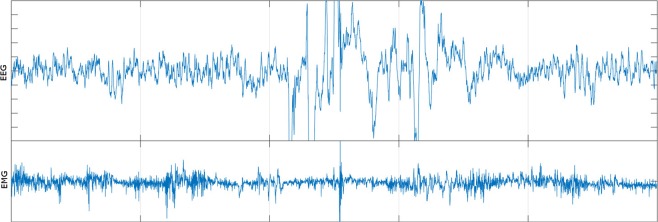
Typical example of noisy signals in large-scale dataset.

In our experiment, the noisiness of biological signals was inspected visually. Due to technical difficulties, we cannot provide quantitative measurements, such as the percentage of noisy parts in each record. Although it is a subjective evaluation, the noisy signals mainly contain noise due to motion artifacts and EMG contamination. Figure 6 shows a typical example of noisy mice signals in the large-scale dataset.

### Performance indices

To evaluate the performance of MC-SleepNet, the following indices are used:

$${{\rm{Recall}}}_{{\rm{s}}}=\frac{{{\rm{E}}}_{({\rm{s}},{\rm{s}})}}{{\sum }_{{\rm{u}}\in {\rm{S}}}{{\rm{E}}}_{({\rm{s}},{\rm{u}})}},\,{{\rm{Precision}}}_{{\rm{s}}}=\frac{{{\rm{E}}}_{({\rm{s}},{\rm{s}})}}{{\sum }_{{\rm{u}}\in {\rm{S}}}{{\rm{E}}}_{({\rm{s}},{\rm{u}})}},\,{\rm{Accuracy}}=\frac{{\sum }_{s\in S}{{\rm{E}}}_{({\rm{s}},{\rm{s}})}}{{\rm{M}}}$$

$$\kappa =\frac{{\rm{Accuracy}}-{p}_{e}}{1-{p}_{e}},\,{p}_{e}=\sum _{s\in S}\,\frac{{\sum }_{{\rm{u}}\in {\rm{S}}}{{\rm{E}}}_{({\rm{s}},{\rm{u}})}}{{\rm{M}}}\cdot \frac{{\sum }_{{\rm{u}}\in {\rm{S}}}{{\rm{E}}}_{({\rm{u}},{\rm{s}})}}{{\rm{M}}},\,(S=\{{\rm{WAKE}},{\rm{Non}}-{\rm{REM}},{\rm{REM}}\}),$$

where *E*_(*s*,*u*)_ is the number of epochs scored as stage *s* and *u* by the experts and MC-SleepNet, respectively. In addition, *M* is the number of all the test epochs. Note that the “correct” sleep stage labels were given by human experts in this evaluation, and the “Accuracy” equals the inter-rater agreement rate between human experts and MC-SleepNet.

Recall_s_ and Precision_s_ are the ratios of correctly scored epochs over a certain set of epochs. For example, Precision_s_ denotes the ratio of correctly scored epochs over the epochs labeled as stage *s* by MC-SleepNet. *κ* (kappa statistic) is the standard accuracy statistic considering the distribution of sleep stages and denotes how much the scoring results are in agreement. In general, when the kappa statistic is over 0.8, it is thought that the scoring results are in nearly perfect agreement.

### Random forest model

In the evaluation experiments, we used the random forest model for comparison. The random forest model consists of feature extraction and scoring phases. First, the EEG and EMG signals are converted into the following six feature values in the feature extraction phase:

$${{\rm{P}}}_{{\rm{EEG}}}=\mathop{\sum }\limits_{i}^{5000}\,|{{\rm{eeg}}}_{{\rm{t}}}|,\,{{\rm{P}}}_{{\rm{EMG}}}=\mathop{\sum }\limits_{i}^{5000}\,|{{\rm{emg}}}_{{\rm{t}}}|,$$

$${\rm{W}}=\mathop{\sum }\limits_{i\mathrm{=1}}^{30}\,||{{\rm{EEG}}}_{{\rm{i}}}||,\,{\rm{D}}=\mathop{\sum }\limits_{i\mathrm{=1}}^{6}\,||{{\rm{EEG}}}_{{\rm{i}}}||,$$

$${\rm{T}}=\mathop{\sum }\limits_{i\mathrm{=7}}^{11}\,||{{\rm{EEG}}}_{{\rm{i}}}||,\,{\rm{E}}=\mathop{\sum }\limits_{i\mathrm{=30}}^{100}||{{\rm{EMG}}}_{{\rm{i}}}||,$$

where eeg_t_ and emg_t_ are the voltage values of EEG and EMG signals at time *t*, respectively. EEG_i_ and EMG_i_ denote the power of frequency components of *i* Hz in EEG and EMG signals, respectively.

Then, the random forest identifies the sleep stages from the extracted features. In the experiments. the number of decision trees, maximum depth of each tree, and maximum number of features were set as 20, 10, and 2, respectively. In this study, the scikit-learn library^23^ was used to implement the random forest model.

### Computer hardware

All experiments were conducted on a computer running CentOS Linux release 7.5.1804, Python version 2.7 and TensorFlow^24^. The hardware components are as follows:

**CPU** Intel(R) Xeon(R) CPU E5-2680 v4 @ 2.40 GHz * 1

**GPU** Tesla P100 PCIe 16 GB * 1

**Memory** 64 GB

## Related Work

### Automated sleep stage scoring methods for mice

Several existing sleep stage scoring methods for mice have been proposed^1–8^. As mentioned above, performance evaluation using the large-scale dataset containing 4,200 mouse records is a contribution of this study. Here, we provide a brief introduction to the existing methods and describe the technical originality of this study.

Although the existing sleep stage scoring methods consist of the feature extraction and scoring phases, the employed techniques/models are different.

Most conventional sleep stage scoring methods employ FFT for extracting frequency-domain features^2,4–7^. For example, FASTER^2^ and MASC^4^ use FFT to extract several frequency components of EEG and EMG signals, which are effective in manual sleep stage scoring. Moreover, the scoring method employing CNN has also been proposed^8^. Please see the SPINDLE section for more details.

To model the relationship between the features and sleep stages, the existing methods employ various classification models, such as nonparametric density estimation clustering^2^, support vector machine^4,6^, LSTM model^5^, and hidden Markov model^7,8^. Generally, the models that can handle time-series data and consider sleep transition rules tend to achieve high scoring accuracy. For example, the LSTM model^5^ achieves scoring accuracy that is almost the same level as the existing state-of-the-art method MASC.

In contrast to these methods, we have adopted a CNN and bi-LSTM for the feature extraction and scoring phases. The CNN can locate the effective features automatically, and bi-LSTM can capture the sleep stage transition rules and relationship between the target epoch and its neighboring epochs. By combining these deep learning models, MC-SleepNet achieves high accuracy and high robustness against individual differences and noise.

In addition, we have also developed a rescoring model to improve the recall of REM. The other existing methods cannot adjust their accuracy according to the purpose of the research. Thus, the rescoring model is another feature of MC-SleepNet.

#### MASC

MASC^4^ is one of the state-of-the-art methods for mice sleep stage scoring, proposed by Suzuki *et al*. in 2017^4^. By using the sleep stages of consecutive neighboring epochs as features and employing the rescoring phase for uncertain epochs, MASC achieves high scoring accuracy of 94.9%.

However, the authors have reported that MASC is weak against noise in EEG and EMG signals^4^. In addition, MASC is not practical for large-scale scoring tasks due to the high computational complexity of the support vector machine, which is employed as a scoring model.

#### SPINDLE

SPINDLE^8^ is a scoring method that employs a CNN for feature extraction and achieves high accuracy of 96.8%. However, they adopted “Artifact” as a new sleep stage and ignored them in the accuracy calculation (including “Artifact”, its accuracy decreases to 88.6%).

Moreover, the number of training samples is too small to train a CNN. They used sleep records obtained from only 4–8 mice/rats. Due to the shortage of training samples, the CNN could not locate the feature of individual differences or noise. Thus, the robustness of SPINDLE against them is quite limited.

Employing a CNN for feature extraction and training it with sufficient training samples are essential to make MC-SleepNet robust against individual differences and noise. This will be the main reason why MC-SleepNet can score sleep stages more accurately than other existing methods.

### Automated sleep stage scoring methods for humans

When we designed MC-SleepNet, we referred to several automated methods for scoring human sleep stages^18,25–27^. In particular, some of our ideas were inspired by “DeepSleepNet” by A. Supratak *et al*.^18^. For example, their model also employs multiple CNN blocks with different filter sizes. However, their purpose and the modeling input/output relationship are quite different from those of MC-SleepNet. In addition, it uses only one-channel EEG signal, while MC-SleepNet uses both EEG and EMG signals.

## Conclusion

In this paper, we have proposed a novel sleep stage scoring method named “MC-SleepNet”, which employs an existing neural network (CNN) and long short-term memory (LSTM) as feature extraction and scoring modules, respectively. MC-SleepNet can automatically locate the effective features that are difficult to extract by handcrafted filters and can model the relationship between features and sleep stages considering sleep stage transition rules. Consequently, MC-SleepNet has achieved both high scoring accuracy and high robustness against individual differences and noise.

The experimental results using the large-scale dataset of 4,200 sleep records of mice achieved an accuracy of 96.6% and *κ* statistic of 0.94. These values are higher than the inter-rater agreement rate among human experts and exceed the accuracy of conventional scoring methods. In addition, we also developed a rescoring model using certainty values of scored stages. Although the “vanilla” MC-SleepNet tends to output fewer REM stage labels, this problem can be resolved by employing the rescoring model.

An important future research issue is the reasoning mechanism to show the rationale for the automatic sleep scoring results. Finding optimal hyperparameter values is another important and interesting topic.

We hope that MC-SleepNet enhances the efficiency and quality of sleep stage scoring and contributes to sleep research.

## Data Availability

The datasets analyzed during the current study are available from the corresponding author on reasonable request.
